# Supplementary material for: Time-series transcriptome analysis identified differentially expressed genes in broiler chicken infected with mixed Eimeria species
Source: Front Genet. 2022 Aug 8;13:886781. doi: 10.3389/fgene.2022.886781 (PMC9393255; doi:10.3389/fgene.2022.886781)
Supplement: Supplementary file 2 [file DataSheet1.ZIP › 7dpi_KEGG.Gsea.1625115961950/KEGG_NON_SMALL_CELL_LUNG_CANCER.html]

Details for gene set KEGG\_NON\_SMALL\_CELL\_LUNG\_CANCER[GSEA]

|  || Dataset | TMM\_7dpi\_gct\_format\_7dpi\_gct\_format.Class\_4dpi.cls #PC\_versus\_NC.Class\_4dpi.cls #PC\_versus\_NC\_repos |
| Phenotype | Class\_4dpi.cls#PC\_versus\_NC\_repos |
| Upregulated in class | 0 |
| GeneSet | KEGG\_NON\_SMALL\_CELL\_LUNG\_CANCER |
| Enrichment Score (ES) | -0.500301 |
| Normalized Enrichment Score (NES) | -1.6173096 |
| Nominal p-value | 0.006442706 |
| FDR q-value | 0.08075631 |
| FWER p-Value | 0.6068 |
Table: GSEA Results Summary

  

Fig 1: Enrichment plot: KEGG\_NON\_SMALL\_CELL\_LUNG\_CANCER      
 Profile of the Running ES Score & Positions of GeneSet Members on the Rank Ordered List

  

| SYMBOL | TITLE | RANK IN GENE LIST | RANK METRIC SCORE | RUNNING ES | CORE ENRICHMENT || 1 | RASSF1 | na | 1321 | 0.424 | -0.0941 | No |
| 2 | HRAS | na | 1342 | 0.420 | -0.0796 | No |
| 3 | TGFA | na | 1901 | 0.287 | -0.1152 | No |
| 4 | CASP9 | na | 2022 | 0.268 | -0.1150 | No |
| 5 | RARB | na | 2792 | 0.151 | -0.1734 | No |
| 6 | MAP2K2 | na | 3435 | 0.070 | -0.2244 | No |
| 7 | ERBB2 | na | 3936 | 0.011 | -0.2657 | No |
| 8 | RAF1 | na | 5142 | -0.116 | -0.3620 | No |
| 9 | MAP2K1 | na | 5188 | -0.120 | -0.3611 | No |
| 10 | FHIT | na | 5463 | -0.145 | -0.3784 | No |
| 11 | PLCG1 | na | 6241 | -0.228 | -0.4346 | No |
| 12 | GRB2 | na | 6686 | -0.274 | -0.4612 | No |
| 13 | AKT3 | na | 7033 | -0.312 | -0.4781 | No |
| 14 | E2F1 | na | 7190 | -0.328 | -0.4786 | No |
| 15 | KRAS | na | 7206 | -0.330 | -0.4671 | No |
| 16 | RXRA | na | 7364 | -0.348 | -0.4669 | No |
| 17 | NRAS | na | 7474 | -0.358 | -0.4622 | No |
| 18 | CDK6 | na | 7686 | -0.382 | -0.4652 | No |
| 19 | PDPK1 | na | 8107 | -0.435 | -0.4836 | Yes |
| 20 | RB1 | na | 8176 | -0.443 | -0.4723 | Yes |
| 21 | AKT1 | na | 8399 | -0.472 | -0.4727 | Yes |
| 22 | PIK3CG | na | 8478 | -0.481 | -0.4608 | Yes |
| 23 | FOXO3 | na | 8568 | -0.494 | -0.4492 | Yes |
| 24 | PRKCA | na | 8704 | -0.513 | -0.4408 | Yes |
| 25 | SOS1 | na | 8869 | -0.533 | -0.4340 | Yes |
| 26 | PIK3R2 | na | 9597 | -0.645 | -0.4700 | Yes |
| 27 | RASSF5 | na | 9758 | -0.667 | -0.4578 | Yes |
| 28 | PRKCB | na | 9867 | -0.685 | -0.4405 | Yes |
| 29 | E2F3 | na | 10031 | -0.717 | -0.4266 | Yes |
| 30 | MAPK3 | na | 10320 | -0.774 | -0.4209 | Yes |
| 31 | PIK3CB | na | 10335 | -0.777 | -0.3923 | Yes |
| 32 | CCND1 | na | 10432 | -0.796 | -0.3697 | Yes |
| 33 | PLCG2 | na | 10697 | -0.853 | -0.3590 | Yes |
| 34 | PIK3CD | na | 10707 | -0.855 | -0.3269 | Yes |
| 35 | STK4 | na | 10730 | -0.861 | -0.2957 | Yes |
| 36 | PIK3CA | na | 10920 | -0.912 | -0.2765 | Yes |
| 37 | PIK3R5 | na | 10941 | -0.918 | -0.2429 | Yes |
| 38 | PIK3R3 | na | 11045 | -0.949 | -0.2151 | Yes |
| 39 | CDKN2A | na | 11228 | -1.009 | -0.1915 | Yes |
| 40 | PIK3R1 | na | 11265 | -1.023 | -0.1553 | Yes |
| 41 | SOS2 | na | 11362 | -1.063 | -0.1225 | Yes |
| 42 | EGFR | na | 11415 | -1.085 | -0.0852 | Yes |
| 43 | BRAF | na | 11724 | -1.264 | -0.0624 | Yes |
| 44 | RXRG | na | 12003 | -2.245 | 0.0006 | Yes |
Table: GSEA details [plain text format]

  

Fig 2: KEGG\_NON\_SMALL\_CELL\_LUNG\_CANCER      
 Blue-Pink O' Gram in the Space of the Analyzed GeneSet

  

Fig 3: KEGG\_NON\_SMALL\_CELL\_LUNG\_CANCER: Random ES distribution      
 Gene set null distribution of ES for **KEGG\_NON\_SMALL\_CELL\_LUNG\_CANCER**
